# The Critical Importance of Recognizing De Winter's T-wave Pattern: A Case of Acute Proximal LAD Occlusion by In-Stent Restenosis

**DOI:** 10.7759/cureus.102984

**Published:** 2026-02-04

**Authors:** Hsiao Chin-Yuan, Wu Keng-Yi

**Affiliations:** 1 Internal Medicine Department, Taichung Armed Forces General Hospital, Taichung, TWN; 2 Cardiology Department, Taichung Armed Forces General Hospital, Taichung, TWN

**Keywords:** acute myocardial infarction, de winter's t-wave, myocardial infarction, revascularization, st-segment elevation

## Abstract

De Winter's T-wave pattern is a high-risk ST-elevation myocardial infarction (STEMI) equivalent signifying acute proximal left anterior descending (LAD) occlusion, characterized by precordial upsloping ST-depression and tall, peaked T-waves. We present a 69-year-old man with chest pain whose electrocardiogram (ECG) showed a classic De Winter's pattern. He rapidly developed acute respiratory failure, and his high-sensitivity troponin I peaked at >27,027 pg/mL. Urgent angiography identified severe in-stent restenosis (ISR) in the proximal-mid LAD as the culprit lesion. This was successfully treated with a drug-coating balloon. This case highlights that De Winter's pattern is a critical finding that must be immediately recognized to prevent delays in emergent reperfusion therapy.

## Introduction

Acute coronary occlusion usually manifests as ST-segment elevation on the electrocardiogram (ECG), necessitating immediate reperfusion therapy. However, certain "ST-elevation myocardial infarction (STEMI) equivalent" patterns signify total or near-total coronary occlusion without classic ST-elevation. De Winter's T-wave pattern is a critical, albeit rare, high-risk ECG finding, estimated to occur in approximately 2% of acute anterior myocardial infarctions associated with proximal left anterior descending (LAD) artery occlusion. It is characterized by upsloping ST-segment depression at the J-point leading into tall, prominent, symmetrical T-waves in the precordial leads.

The primary danger of the De Winter pattern lies in its deceptive appearance. Because it lacks typical ST-elevation, it is frequently misdiagnosed as non-ST-elevation acute coronary syndrome (NSTE-ACS), potentially leading to delayed treatment. However, current guidelines recognize this pattern as an indication for emergent reperfusion, identical to the management of classic STEMI. Failure to promptly recognize this "clinical trap" can result in extensive myocardial damage and adverse outcomes. We report a case of a patient presenting with De Winter's pattern caused by severe in-stent restenosis (ISR), highlighting the necessity for rapid identification and intervention.

## Case presentation

A 69-year-old man presented to the emergency department (ED) on June 12 with a chief complaint of severe chest pain persisting for hours. He had initially visited the ED earlier that same day for dizziness, at which time his electrocardiogram (ECG) showed sinus rhythm with nonspecific T-wave abnormalities in the inferior leads, and he was discharged after symptom improvement. However, he returned later that morning with progressive dyspnea and chest pain. Upon arrival, his vital signs revealed a blood pressure of 149/70 mmHg, a heart rate of 82 bpm, and a respiratory rate of 16 breaths/minute. A physical examination was notable for bilateral wheezing. The patient's past medical history was significant for triple-vessel coronary artery disease (TVD). His history included a prior percutaneous coronary intervention (PCI) with a 2.75×33 mm bare-metal stent (BMS) placed in the mid-left anterior descending (LAD) artery, as well as plain old balloon angioplasty (POBA) of the mid-to-distal left circumflex (LCX) artery. Additionally, he had a known chronic total occlusion (CTO) of the proximal right coronary artery (p-RCA) and suffered from chronic congestive diastolic heart failure with a New York Heart Association (NYHA) functional classification of III.

Key investigations

**Figure 1:**
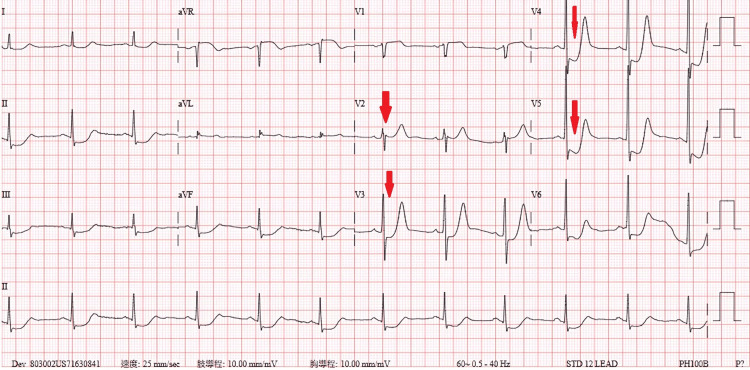
Initial 12-lead ECG obtained during chest pain The tracing displays the classic De Winter's T-wave pattern, characterized by upsloping ST-segment depression at the J-point leading into tall, symmetrical, peaked T-waves in precordial leads V2-V5 (red arrow). This pattern is a STEMI equivalent indicating acute proximal LAD artery occlusion. ECG: electrocardiogram, LAD: left anterior descending, STEMI: ST-elevation myocardial infarction

**Figure 2:**
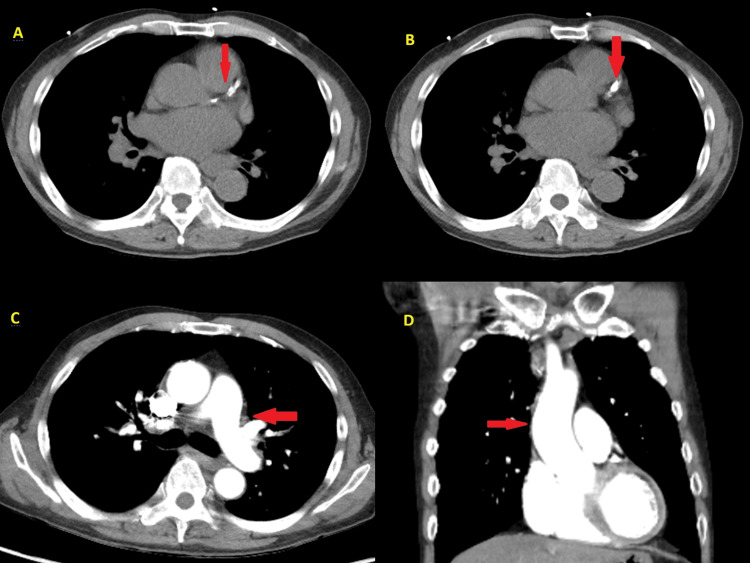
Contrast-enhanced chest CT (A and B) Axial views demonstrate significant coronary artery calcification (red arrows). (C and D) Visualization of the aortic arch confirms the absence of an intimal flap or aortic dissection. CT: computed tomography

**Figure 3:**
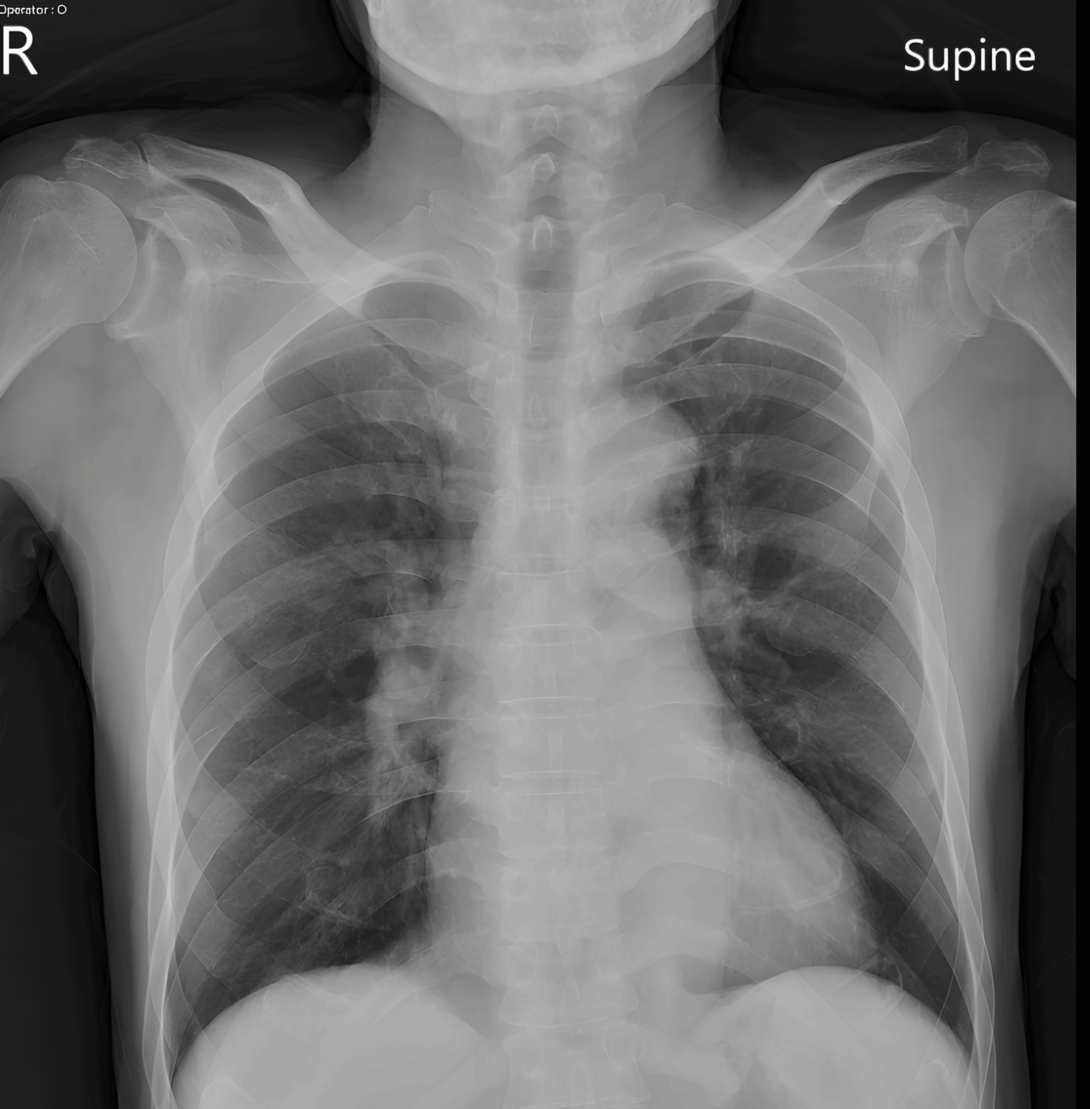
Chest radiograph on presentation The bilateral lung fields appear relatively clear without evidence of significant pulmonary edema, vascular congestion, or active consolidation. The cardiomediastinal silhouette and costophrenic angles are unremarkable.

A 12-lead ECG (Figure 1) revealed sinus rhythm with prominent, upsloping ST-segment depression at the J-point, most notable in the precordial leads V2 through V5. These ST depressions were immediately followed by tall, positive, and symmetrical T-waves, particularly in leads V2-V4. This constellation of findings is consistent with the classic De Winter's T-wave pattern, an ST-segment elevation myocardial infarction (STEMI) equivalent indicating an acute occlusion of the proximal LAD artery. Initial laboratory results were markedly abnormal, with a high-sensitivity troponin I level of 5,306.9 pg/mL(normal range: 0-40 pg/mL), a D-dimer level of 4,127.43 ng/mL (normal range: <500 pg/mL), and a B-type natriuretic peptide (BNP) level of 210 pg/mL (normal range: <100 pg/mL). To exclude aortic dissection, given the severe chest pain, a chest computed tomography (CT) scan was performed, which revealed atherosclerosis with calcification in the coronary arteries, but no dissection (Figure 2). A chest X-ray taken at the time showed no signs of pulmonary edema (Figure 3).

Hospital course and management

Initially, the absence of classic ST-segment elevation led to a working diagnosis of NSTE-ACS (unstable angina) and acute decompensated heart failure. Consequently, the patient was admitted to the intensive care unit (ICU) for conservative medical management. This initial classification highlights the deceptive nature of the De Winter pattern, which risks being overlooked as a non-emergent condition despite representing a critical coronary occlusion.

However, the clinical course rapidly deteriorated. At midnight on June 13, the patient developed severe dyspnea at rest with progressive oxygen desaturation, advancing to acute respiratory failure that necessitated emergency endotracheal intubation and mechanical ventilation. Subsequent cardiac biomarkers confirmed a massive myocardial infarction; his high-sensitivity troponin I rose to >27,027 pg/mL (the upper limit of detection), and creatine phosphokinase (CPK) peaked at 1,953 U/L (normal range: 25-190 U/L).

**Figure 4:**
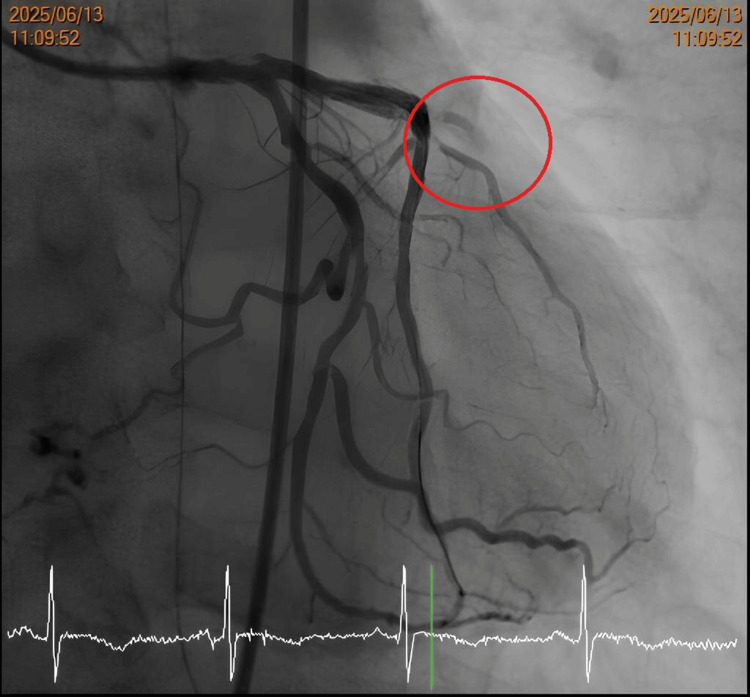
Pre-intervention coronary angiography The angiogram reveals a critical lesion identified as severe ISR in the proximal-to-mid LAD artery (red circle), causing significant flow limitation. ISR: in-stent restenosis, LAD: left anterior descending

**Figure 5:**
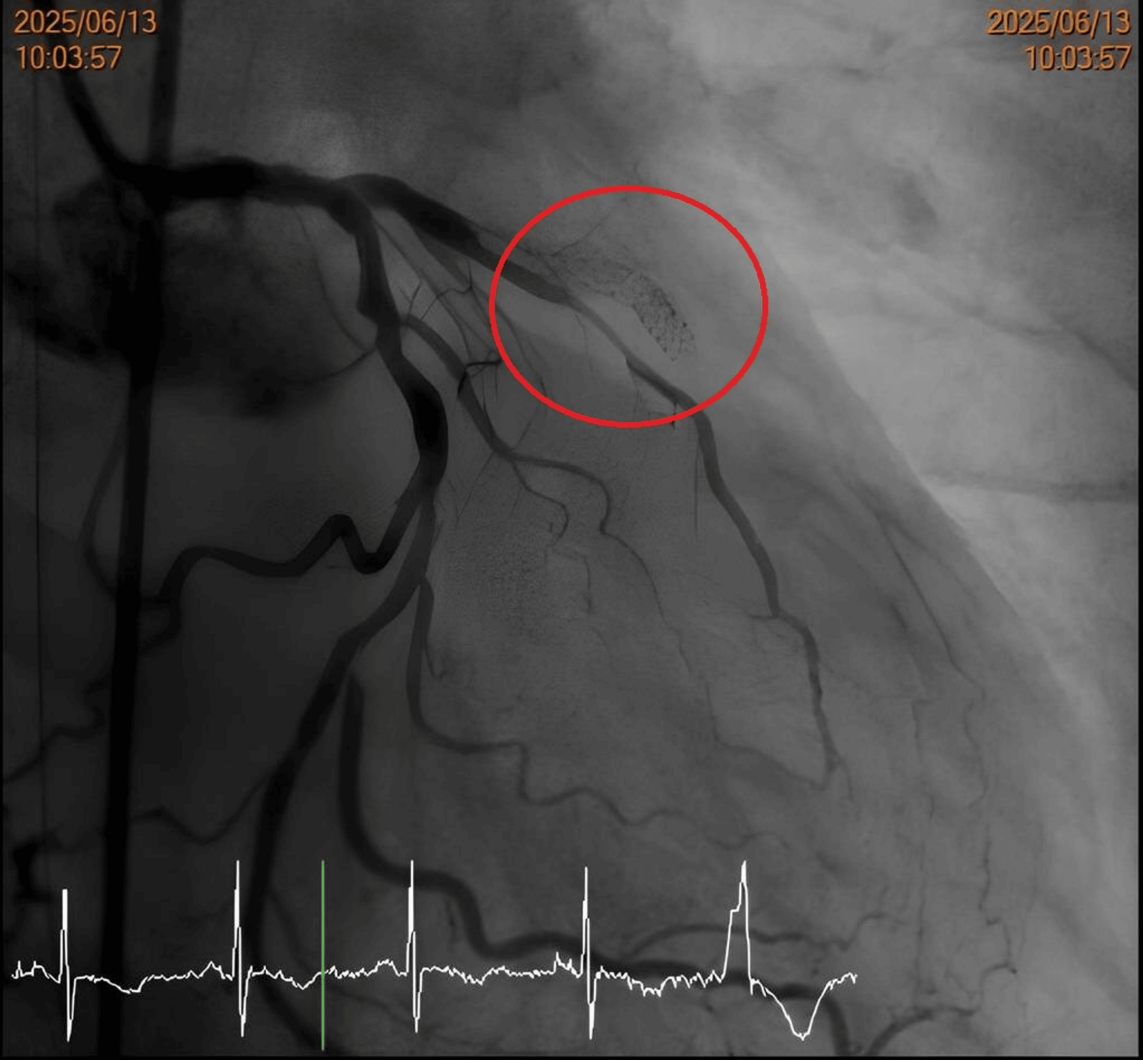
Post-intervention coronary angiography Following treatment with a DCB, the previously stenotic segment in the LAD artery is widely patent (red circle), demonstrating successful restoration of blood flow. DCB: drug-coated balloon, LAD: left anterior descending

Recognizing the high-risk combination of De Winter's ECG pattern and the patient's history of a prior LAD stent, the clinical team suspected catastrophic in-stent restenosis (ISR) and immediately escalated management to emergent reperfusion. The patient underwent urgent coronary angiography on June 13, approximately 25 hours after his initial ED presentation. The angiogram confirmed triple-vessel disease (Figure 4) and identified the culprit lesion in the LAD. Intravascular ultrasound (IVUS) assessment revealed the precise mechanism of occlusion: a calcified nodule with plaque protrusion within the prior stent, alongside evidence of stent under-expansion. The ISR lesion was treated via serial balloon angioplasty using multiple balloons (ranging from 1.2 mm to 3.0 mm non-compliant balloons) to adequately prepare the calcified bed. This was followed by the application of a 3×40 mm drug-coated balloon (Selution SLR; MedAlliance, Nyon, Switzerland) at 14 atm to inhibit future restenosis. The procedure concluded successfully, with final angiography demonstrating restoration of TIMI 2-3 flow in the distal LAD (Figure 5).

Post-intervention Course

**Figure 6:**
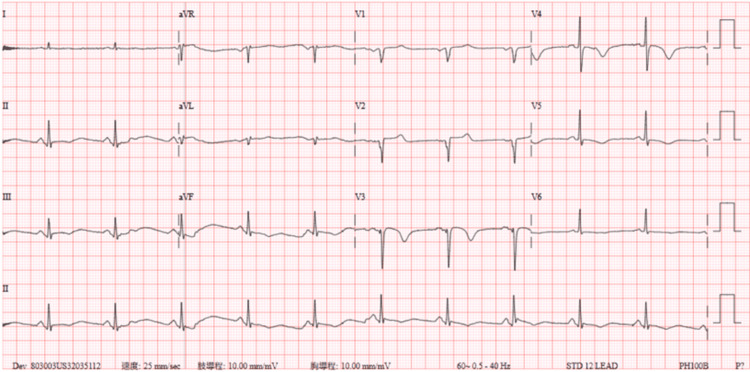
Post-intervention 12-lead ECG The tracing demonstrates sinus rhythm with evolutionary changes of a recent anterior myocardial infarction. Key findings include deep Q-waves in leads V1-V3 and widespread, deep T-wave inversions in leads V2-V6, consistent with the post-reperfusion state. ECG: electrocardiogram

The following is the ECG from the day after the cardiac catheterization (Figure 6). The patient's hospital course was complicated. He developed a high-grade fever (up to 39°C) post-procedure. On June 16, a workup revealed he was positive for the influenza A virus, for which he was treated with antiviral medication. He had a prolonged ICU stay and was eventually weaned from the ventilator and transferred to the general ward on June 20. His course was also complicated by drowsiness, requiring a psychiatric consultation, and a recurrent fever in early July, which was treated with empiric antibiotics.

After his medical condition stabilized, he was ready for discharge. On July 8, 2025, he was transferred to a nursing home for continued care.

## Discussion

This case illustrates De Winter's T-wave pattern, a critical STEMI equivalent indicating acute proximal left anterior descending (LAD) artery occlusion. First described by de Winter et al., this pattern occurs in approximately 2% of acute LAD occlusions and is characterized by a 1-3 mm upsloping ST-segment depression at the J-point in precordial leads V1-V6, transitioning into tall, symmetrical T-waves, frequently accompanied by slight ST-elevation (0.5-1 mm) in lead aVR [1].

In our patient, the ECG (Figure 1) exhibited these classic features in leads V2-V5, which were correctly flagged by automated analysis as "severe global ischemia," reflecting the massive myocardial territory at risk. This pattern is considered dynamic and may represent a "pre-infarction" state that can evolve into classic STEMI or alternate with Wellens' syndrome [2,3]. Regarding the underlying electrophysiological mechanism, while it remains a subject of discussion, leading theories suggest that the upsloping ST depression represents extensive subendocardial ischemia, while the tall, peaked T-waves may stem from sarcolemmal ATP-sensitive potassium (K-ATP) channel dysfunction [4,5]. Regardless of the ionic mechanism, this pattern signifies a critical occlusion myocardial infarction (OMI), a classification that aligns with the recent paradigm shift away from strict STEMI/NSTEMI definitions [6,7]. In this specific patient, the acute occlusion was caused by severe in-stent restenosis (ISR) within a previously placed bare-metal stent.

The primary danger of the De Winter pattern lies in its potential for misinterpretation as NSTE-ACS, leading to critical delays in reperfusion [1]. However, current guidelines, including the 2021 AHA/ACC Guideline for the Evaluation and Diagnosis of Chest Pain, explicitly recognize the De Winter pattern as an ECG equivalent of acute coronary occlusion. Consequently, these patients should be managed with the same urgency as classic STEMI, proceeding directly to emergent reperfusion therapy. Although our patient's diagnosis was initially delayed, rapid clinical decompensation and profound biomarker elevation (high-sensitivity troponin I > 27,027 pg/mL) eventually triggered successful intervention with balloon angioplasty and a drug-coating balloon (DCB). This case reinforces the principle that the De Winter ECG pattern itself should serve as the primary trigger for emergent catheterization, even before clinical collapse or biomarker confirmation.

## Conclusions

This case demonstrates that De Winter's T-wave pattern, characterized by precordial upsloping ST-segment depression with tall, symmetrical T-waves, is a critical sign of acute proximal LAD occlusion. However, as illustrated in this patient, the absence of classic ST-segment elevation can lead to initial misinterpretation as NSTE-ACS, resulting in dangerous delays in reperfusion. Therefore, immediate recognition of this pattern as a high-risk STEMI equivalent is essential to trigger emergent catheterization and prevent catastrophic clinical deterioration.
